# Metadehumanization and Self-dehumanization are Linked to Reduced Drinking Refusal Self-Efficacy and Increased Anxiety and Depression Symptoms in Patients with Severe Alcohol Use Disorder

**DOI:** 10.5334/pb.1058

**Published:** 2021-07-26

**Authors:** Sullivan Fontesse, Stéphanie Demoulin, Florence Stinglhamber, Philippe de Timary, Pierre Maurage

**Affiliations:** 1 Louvain Experimental Psychopathology Research Group, Psychological Sciences Research Institute, UCLouvain, Place C. Mercier 10, B-1348 Louvain-la-Neuve, Belgium; 2 Psychological Sciences Research Institute, UCLouvain, Place C. Mercier 10, B-1348 Louvain-la-Neuve, Belgium; 3 Work and Organizational Psychology Lab, Psychological Sciences Research Institute, UCLouvain, Place C. Mercier 10, B-1348 Louvain-la-Neuve, Belgium

**Keywords:** interpersonal treatment, psychiatry, infrahumanization, discrimination, substance

## Abstract

Metadehumanization, the perception of being treated as less than a human by others, is a pervasive phenomenon in intergroup relations. It is dissociated from stigmatization or stereotypes, and it has been recently identified as a critical process in severe alcohol use disorders (SAUD). Metadehumanization is associated with a wide array of negative consequences for the victim, including negative emotions, aversive self-awareness, cognitive deconstruction, and psychosomatic strains, which are related to anxiety and depression. This study aims to investigate if metadehumanization occurring among patients with SAUD is associated with clinical factors involved in the maintenance of the disease, namely symptoms of depression or anxiety and drinking refusal self-efficacy. A cross-sectional study was conducted among 120 patients with SAUD. Self-reported questionnaires measured metadehumanization, self-dehumanization (i.e., the feeling of being less than a human), anxiety, depression, drinking refusal self-efficacy, and demographics. Metadehumanization was significantly associated with self-dehumanization, anxiety, depression, and drinking refusal self-efficacy. Additionally, path analyses showed that self-dehumanization mediated the links between metadehumanization and clinical variables. These results indicate that metadehumanization and self-dehumanization could be essential factors to consider during SAUD treatment, as they are associated with increased psychiatric symptoms and reduced drinking refusal self-efficacy.

## Introduction

Dehumanization, corresponding to the denial of other individuals’ humanity, is based on the refutation of uniquely or essentially human characteristics (e.g., civility, refinement, moral sensibility, emotional responsiveness, interpersonal warmth, or cognitive openness; Haslam, 2006). Dehumanization is distinct from stigmatization (defined as a negative taint applied to some groups; Goffman, 1963), as dehumanization is specifically the reduced attribution of humanity. Dehumanization is present in extreme situations such as genocides or long-lasting violent conflicts (Kelman, 1973; Kteily et al., 2016). However, milder forms of dehumanization are also part of everyday life when people are neglected or maltreated (Leyens et al., 2001; Bastian & Haslam, 2010).

### Metadehumanization

Based on the definition of dehumanization, metadehumanization can be defined as the subjective perception of being considered by others as lacking uniquely or essentially human characteristics (Bastian & Haslam, 2011). Metadehumanization has been defined as ‘this perception that one’s own group is perceived by another as less than fully human’ and ‘the degree to which people believe that a target group denies humanity to their own’ (Kteily et al., 2016; Kteily & Bruneau, 2017). Metadehumanization is thus a metacognitive process as it rests on the processing of what others think about one’s group. However, just as dehumanization can target an individual or a group (Leyens, 2009; Gwinn et al., 2013; Trifiletti et al., 2014), we argue that one can experience metadehumanization toward his/her group or himself/herself.

Maltreatments that can induce metadehumanization comprise many different situations such as being ostracized, betrayed, treated as immoral, treated instrumentally, or humiliated (Bastian & Haslam, 2011). For example, in real-life situations, a client completely ignored by a cashier or an employee belittled and yelled at by his/her boss might feel dehumanized. Metadehumanization provokes adverse outcomes (Bastian & Haslam, 2011; Bastian & Crimston, 2014; Caesens et al., 2017; Zhang et al., 2017; Nguyen & Stinglhamber, 2018) such as negative emotions (sadness, anger, and guilt), aversive self-awareness, cognitive deconstruction, and psychosomatic strains (sleeping trouble, headache, heartburn, eyestrain, loss of appetite, dizziness, and fatigue). People who feel dehumanized also tend to dehumanize others in return (Kteily et al., 2016; Bruneau & Kteily, 2017), which is detrimental to their social interactions, as dehumanizing someone else can lead to negligence, maltreatments, and violent behaviors (Bandura, 1999; Kteily et al., 2015).

### Metadehumanization and self-dehumanization

An individual feeling dehumanized by others can interiorize this dehumanizing perspective in his/her self (i.e., develop self-dehumanization, Bastian & Crimston, 2014). Whereas metadehumanization is the perception of being dehumanized by others, self-dehumanization is the self-perception of being less human than others. In this case, one thus perceives himself/herself as less than a human through the denial of uniquely or essentially human characteristics (e.g., maturity, refinement). Just as self-stigma is the internalization of stigma awareness and thus results from it, we argue that self-dehumanization is the internalization of metadehumanization and could thus result from it (Schomerus et al., 2011). Theoretically, self-dehumanization could be more problematic than metadehumanization because it denotes a more advanced internalization process, as metadehumanization is the awareness of being dehumanized even if the victim does not agree with this perception nor apply it to its self-perception. Self-dehumanization could thus lead to stronger negative consequences. However, metadehumanization and self-dehumanization are rarely studied together (but see Bastian & Haslam, 2011).

### Metadehumanization, severe alcohol-use disorders, and other psychopathological symptoms

Until recently, metadehumanization had not been investigated in patients with psychiatric disorders, despite dehumanization described as endemic to medicine (Haque & Waytz, 2012). A theoretical proposal suggested that patients with severe alcohol use disorders (SAUD) could be particularly dehumanized by others, which would be detrimental to their mental health (Fontesse et al., 2019). The first empirical evidence of metadehumanization in patients with psychiatric disorders has been offered by a recent study among patients with SAUD (Fontesse et al., 2020), revealing that these patients present strong metadehumanization feelings, which are linked to fundamental needs threat, reduced self-esteem, decreased use of functional coping strategies, and increased use of dysfunctional ones, including alcohol use. Interestingly, all these factors are associated with more intense SAUD; metadehumanization could thus be a vulnerability factor regarding SAUD severity (Fontesse et al., 2020).

However, a still unaddressed question is whether metadehumanization could also be an aggravating factor regarding other psychiatric symptoms frequently observed in SAUD and linked to the aggravation of such disorders. Indeed, metadehumanization is linked to multiple symptoms of depressive disorders such as sadness, guilt, loss of appetite, and fatigue (Bastian & Haslam, 2011; Caesens et al., 2017). The same goes for anxiety disorders symptomatology because sleep disturbance, tiredness, and other psychosomatic strains are known consequences of metadehumanization (Caesens et al., 2017). Metadehumanization can also lead to cognitive consequences like cognitive deconstruction, manifested through attentional difficulties (Bastian & Haslam, 2011; Caesens et al., 2017). Metadehumanization might thus be linked not only to SAUD severity but also to symptoms of other psychological disorders such as anxiety and depression. These states are frequently observed in SAUD (Davidson, 1995; Spangenberg & Campbell, 1999; Grant et al., 2004) and can impede abstinence (Driessen et al., 2001). Indeed, after being treated for SAUD, patients with anxiety disorders are twice more likely to relapse (Kushner et al., 2005); patients who present both anxiety and depressive disorders are four times more likely to relapse (Driessen et al., 2001). Finally, patients suffering from SAUD who present depressive or anxiety disorders are also more likely to attempt suicide (Driessen et al., 1998; Richa et al., 2008). As a whole, these psychopathological comorbidities constitute critical factors in SAUD maintenance. The first goal of this paper is thus to investigate the associations between metadehumanization, depression, and anxiety disorders in SAUD.

Furthermore, self-dehumanization has never been measured in patients with psychiatric disorders. Thus, the second goal of this study is to address this shortcoming by integrating metadehumanization and self-dehumanization in the same study. Namely, following the reasoning exposed above and previous research showing that “dehumanization seeps into the self-perception of the victims” (Bastian & Crimston, 2014), we argue that metadehumanization precedes self-dehumanization; self-dehumanization might thus mediate the links between metadehumanization and other dependent variables such as anxiety and depression.

As previously stated, SAUD patients with comorbidities often present heavier forms of dependence and are harder to treat. We propose that metadehumanization, through self-dehumanization, is not only associated with symptoms of psychopathological disorders such as anxiety and depression, but also changes patients’ drinking-refusal self-efficacy. Dehumanization has been repeatedly linked to reduced perception of competence, self-restraint and control (Haslam, 2006; Li et al., 2014). We argue that self-dehumanization should be associated with similar perceptions but directed toward the self instead of others (i.e., reduced self-perception of competence, self-restraint, and control). We thus hypothesize metadehumanization and self-dehumanization to be linked to reduced drinking refusal self-efficacy. Furthermore, drinking refusal self-efficacy will provide a proxy of patients’ relapse risk. Indeed, it is linked to dependence severity, the quantity of alcohol consumed, and the frequency of alcohol consumption (Connor et al., 2000; Connor et al., 2008). Drinking refusal self-efficacy has also been repeatedly linked to problem drinking and alcohol-related consequences in non-clinical samples (Ehret et al., 2013; Klanecky et al., 2015). Moreover, when facing normative pressure to consume alcohol, people with high drinking refusal self-efficacy report less intention to drink alcohol than people with low drinking refusal self-efficacy (Jang et al., 2013). If patients present a lower level of drinking refusal self-efficacy, they are thus more at risk of relapse.

To sum up, multiple factors known for their importance in SAUD prognosis will be investigated: metadehumanization, self-dehumanization, anxiety, depression, and drinking refusal self-efficacy. We expect that higher levels of metadehumanization would be linked to higher levels of depression, anxiety, and lower drinking refusal self-efficacy in patients with SAUD. Because self-dehumanization is theorized as a more advanced step in the internalization of dehumanization, we propose that the links observed between metadehumanization and the dependent variables will be explained by self-dehumanization as it should be closer to the negative factors associated with metadehumanization.

## Methods

### Participants

One hundred and twenty inpatients undergoing alcohol detoxification treatment were recruited. Psychiatrists selected patients free from other important medical problems and neurological diseases. Patients with SAUD meeting our criteria were recruited after at least 14 days of abstinence. As ten participants did not complete the second part of the survey (i.e., measures of self-dehumanization, drinking refusal self-efficacy, depression, and anxiety), they were removed from our analyses. Analyses were thus conducted on 110 participants (30 females, 80 males). Participants had a mean age of 48.3 years (S.D. = 10.9), most of them had a high school diploma or lower (52,9%). Participants consumed 19.60 (S.D. = 12.45) units of alcohol per day before detoxification. Patients had been suffering from SAUD for 13.6 years on average (S.D. = 10.88) and had been involved in 2.66 (S.D. = 3.24) past alcohol detoxification treatments.

### Materials

The survey measured metadehumanization, self-dehumanization, anxiety, depression, drinking refusal self-efficacy, and demographics. This study is part of a larger project exploring the emotional and cognitive correlates of SAUD.

#### Metadehumanization

A self-reported metadehumanization 13-item scale (Cronbach’s α = .93) measured how participants felt dehumanized by society (e.g., ‘As an alcohol-dependent person, society treats me as a sub-evolved being,’ ‘[…] as an immature person’, ‘[…] as someone lacking emotions’, ‘[…] as an automata’, ‘[…] as an object’, see Supplementary Material 1 for the full scale). This scale focuses on participants’ perception of being dehumanized by society. The scale was adapted from previous work on organizational dehumanization, a form of metadehumanization where the dehumanizer is one’s organization (Caesens et al., 2017). The scale of organizational dehumanization was inspired by previous work (Haslam, 2006). It thus encompasses known criteria of dehumanization, such as immaturity, superficiality, and coldness. Agreement with the items was measured using a 7-point Likert-type scale (from ‘Completely disagree’ to ‘Completely agree’). Answers were averaged to compute a mean score ranging from 1 to 7.

#### Self-dehumanization

Participants’ self-dehumanization feelings were measured with 13 items (α = 0.79). This scale was adapted from the metadehumanization scale to refer to self-related feelings (e.g., ‘As an alcohol-dependent person, I sometimes consider myself as a sub-evolved being,’ ‘[…] as an immature person’, ‘[…] as someone lacking emotions,’ ‘[…] as an automaton,’ ‘[…] as an object,’ see Supplementary Material 2 for the full scale). The items were close to those presented in the metadehumanization scale (they were adapted to measure self-perceptions); the order of the items was different. Agreement with the items was measured using a 7-point Likert-type scale (from ‘Completely disagree’ to ‘Completely agree’). Answers were averaged to compute a mean score ranging from 1 to 7.

#### State-anxiety

State-anxiety was measured using a 20-item French scale (α = 0.96) adapted from the State-Trait Anxiety Inventory form Y (STAI-Y; Spielberger, 1983; Gauthier & Bouchard, 1993). Agreement with the items was measured using a 4-point Likert-type scale (from ‘No’ to ‘Yes’). Answers were summed to compute a total score (range: 20–80).

#### Depression

The Beck Depression Inventory-short version (BDI, α = 0.84) was used to assess participants’ levels of depression with 13 items (Beck et al., 1996; Luty & O’Gara, 2006). Items on this multiple answers scale were scored from 0 to 3. Answers were summed to compute a total score (range: 0–39).

#### Drinking refusal self-efficacy

Participants’ self-perceived ability to resist alcohol was assessed using the 19-item Drinking Refusal Self-Efficacy Questionnaire-Revised (DRSEQ-R; Oei et al., 2005). Scale anchors were ‘I am sure I would drink,’ ‘I would probably drink,’ ‘I might drink,’ ‘I might not drink,’ ‘I would probably drink,’ ‘I am sure I would not drink.’ We computed a general mean drinking refusal self-efficacy score (α = 0.97, range: 1–6).

### Procedure

The study was conducted in six hospitals between September 2016 and June 2018. Patients were recruited during their detoxification stay, and they received a full written description of the study. All participants were informed that they could not be identified via our scientific communications as we fully anonymized them. Participants answered this survey and other questionnaires from a much larger project in two one-hour sessions placed on two different days of the same week. Metadehumanization was assessed on the first session; other variables were assessed on the second one. Participants answered the survey in groups. An experimenter was present to answer their questions. The study protocol was approved by a biomedical committee of the University (B403201732246) and respected the Declaration of Helsinki, as revised in 2008. All patients provided written informed consent. The data used in this paper was extracted from the same large database used in Fontesse et al. (2020). Participants’ responses on the metadehumanization and self-dehumanization scales have thus been reused. However, all the relations with outcomes investigated in this paper are completely original.

### Statistical analyses

SPSS 25 was used for descriptive statistics and correlations. The classical .05 p-value was used as the threshold for statistical significance. StataSE 15 was used to conduct the path-analysis model, which allows for complex model testing. The path-analysis model was estimated using maximum likelihood with missing values, and standardized path coefficients are reported (Wright, 1934). Direct and indirect effects were also tested with StataSE 15.

## Results

### Descriptive statistics and correlations

Anxiety scores indicate that 44% of our sample express a very low level of anxiety (STAI < 36; Spielberger 1983), 25% a low level (35 < STAI < 46), 15% an average level (45 < STAI < 56), 8% a high level (55 < STAI < 66), and 7% a very high level of anxiety (STAI > 65; see ***Table 1*** for the mean, standard deviation, minimum and maximum of all scales). For depression, BDI scores indicate no depressive symptoms for 26% of our sample (BDI < 5; Beck et al. 1996), mild depressive symptoms for 17% (4 < BDI < 8), moderate for 39% (7 < BDI < 16) and severe for 17% (BDI > 15). All correlations between variables of interest are presented in ***Table 1***.

**Table 1:** Descriptive statistics, Cronbach alphas, and correlations between experimental variables.


	*M*	*SD*	Min-Max	1.	2.	3.	4.	5.

1. Metadehumanization	3.20	1.42	1–6.54	(0.93)				

2. Self-dehumanization	2.86	1.06	1–6	0.45***	(0.86)			

3. Anxiety	39.36	14.34	20–78	0.27**	0.48***	(0.96)		

4. Depression	9.11	5.90	0–24	0.21*	0.47***	0.74***	(0.84)	

5. Drinking refusal self-efficacy	4.17	1.35	1.05–6	–0.25**	–0.35**	–0.38***	–0.35***	(0.97)


*Note: N* = 110. Cronbach alphas are between brackets on the diagonal. * p < 0.05; ** p < 0.01; *** p < 0.001.

### Path-analysis model

Using path analysis, a model [χ^2^(2) = 14.34; RMSEA = 0.24; CFI = 0.89] testing only the direct links between metadehumanization and the three dependent variables (without self-dehumanization) and considering the covariance between anxiety and depression revealed that metadehumanization was significantly associated with anxiety (*γ* = 0.27, p = 0.002), depression (*γ* = 0.21, p = 0.018), and drinking refusal self-efficacy (*γ* = –0.25, p = 0.005).

**Figure 1:**
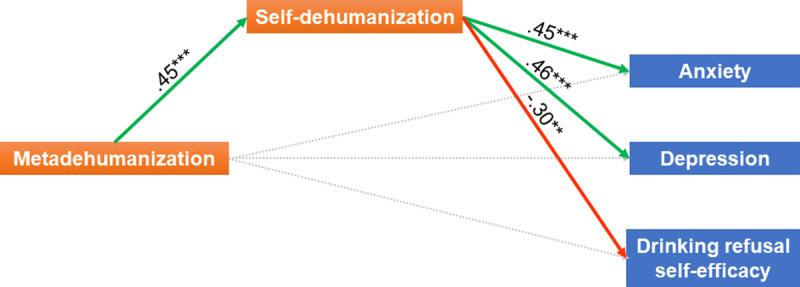
Statistical model tested [χ^2^(2) = 7.893; RMSEA = 0.16; CFI = 0.96]. Significant standardized regressions paths are depicted as large arrows; non-significant paths are depicted as dotted lines. Covariances, not depicted, were entered between anxiety and depression residuals, as they are closely related (r = 0.74, p = 0.000). * p < 0.05; ** p < 0.01; *** p < 0.001.

When entering self-dehumanization in the model [χ^2^(2) = 7.893; RMSEA = 0.16; CFI = 0.96] as a mediator between metadehumanization and the dependent variables, metadehumanization was positively linked to self-dehumanization (γ = 0.45, p = 0.000; ***Figure 1***) and the other links became non-significant (anxiety: *γ* = 0.06, p = 0.487; depression: *γ* = 0.00, p = 0.98; drinking refusal self-efficacy: *γ* = –0.11, p = 0.266). Furthermore, self-dehumanization was positively associated with anxiety (β = 0.45, p = 0.000), depression (β = 0.46, p = 0.000), and negatively associated with drinking refusal self-efficacy (β = –0.30, p = 0.002). All indirect effects of metadehumanization through the mediator, self-dehumanization, were found to be significant: on anxiety (indirect effect = 0.20, p = 0.000), depression (indirect effect = 0.21, p = 0.000), and drinking refusal self-efficacy (indirect effect = –0.14, p = 0.009).

## Discussion

This study investigated the links between metadehumanization, self-dehumanization, and drinking refusal self-efficacy as well as symptoms of anxiety and depression in patients with SAUD. Our results offered key insights related to dehumanization in patients with a psychiatric disorder, respectively related to the links between metadehumanization and clinical outcomes and to the mediating role of self-dehumanization.

### Metadehumanization and symptoms of mood disorders

First, metadehumanization is related to more symptoms of anxiety and depression and to lower levels of drinking refusal self-efficacy. Patients who experienced higher levels of metadehumanization may have reduced ability to maintain abstinence, as symptoms of anxiety and depression and reduced drinking refusal self-efficacy are linked to increased relapse risk (Driessen et al., 2001; Kushner et al., 2005; Gullo et al., 2010). This finding highlights the need to consider interpersonal factors in the emergence of psychological disorders. The proposal that social variables should be considered, beyond disease-related and personal characteristics, has a long history in psychiatry. Indeed, Philippe Pinel (1806) already identified humanitarian care, benevolent support, and encouragement as primordial steps toward the recovery of patients with psychiatric disorders. More recent paradigms such as the social perspectives of psychopathological disorders also identify social determinants (e.g., poverty, unemployment, and discrimination) as causes of psychopathology (Albee, 1982), a view also endorsed by the World Health Organization (Marmot et al., 2012; World Health Organization Regional Office for Europe, 2014). Such paradigms call for the acknowledgment that emotional distress and mental disturbances can be caused by dehumanizing social influences (Albee, 1982), which opens new avenues for primary prevention (Carod-Artal, 2017).

### Self-dehumanization

The second main finding of our study is that self-dehumanization is a crucial process in our model. Indeed, metadehumanization and self-dehumanization are linked, congruent with the theoretical proposal that metadehumanization might reinforce self-dehumanization (although the reverse is also possible). The perception of being dehumanized by others is thus linked to the self-perception of being less than human. Furthermore, self-dehumanization mediated all the links observed between metadehumanization and measured outcomes. For patients with SAUD, interiorizing other people’s dehumanizing perspective into their self-perspective was associated with increased anxiety, increased depression, and decreased drinking refusal self-efficacy. When controlling for self-dehumanization, metadehumanization was not directly associated with anxiety, depression, and drinking refusal self-efficacy anymore. Instead, metadehumanization was associated with these dependent variables through self-dehumanization. In other words, what is most important regarding dehumanization might not be the metadehumanization *per se* but instead how metadehumanization is integrated into patients’ self-perspectives (i.e., how they self-dehumanize).

Self-dehumanization has been linked to negative emotions (shame, guilt, sadness, and anger), aversive self-awareness, and cognitive deconstructive states (Bastian & Crimston, 2014). Negative emotions can provoke lapses in self-regulation, which in turn can lead to relapse (Heatherton & Wagner, 2011). Aversive self-awareness leads people to a state of cognitive deconstruction characterized by biased focalization on the present and neglect of long-term consequences (Twenge et al., 2003; Heatherton & Wagner, 2011). These two mechanisms could explain why people who suffer from addictive states can relapse by ignoring the long-term consequences of their actions in an attempt to escape aversive self-awareness. This proposal has some empirical support, as consuming alcohol decreases self-awareness, especially among individuals with high self-consciousness (Hull, 1981). Overall, our results should warrant researchers’ attention to self-dehumanization, which should be studied in addictive disorders.

### Clinical implications

Our first key result, showing that metadehumanization is linked to increased psychopathological symptoms and reduced drinking refusal self-efficacy, emphasizes the need to improve how patients are treated. While reducing stigma against people with SAUD and other psychiatric disorders is already an important topic (Corrigan et al., 2017; Melchior et al., 2019), reducing dehumanization has not received considerable attention. Dehumanization and stigma are interrelated interpersonal treatments (heavily stigmatized targets tend to be dehumanized; Harris & Fiske, 2006; Cameron et al., 2016), but they are also distinct and dissociable theoretically and empirically (Bruneau & Kteily, 2017). Interventions aimed at improving how patients with SAUD or other psychiatric disorders are treated in our societies should also be developed to improve humanity attribution toward these patients. Improving society’s perception of patients with psychiatric disorders’ human attributes (e.g., interpersonal warmth, moral restraint, maturity), improving their humanization, and creating opportunities for positive contacts between patients with psychiatric disorders and others could serve this purpose (Capozza et al., 2013). Reducing dehumanization toward patients with SAUD, and thus their metadehumanization, could positively impact their prognosis and well-being.

In addition to interventions on metadehumanization, interventions on self-dehumanization could also be developed. The pattern of associations found in this study emphasizes both the importance of perception from others (through metadehumanization) and self-perceptions (through self-dehumanization) in relation to psychopathological symptoms in patients with SAUD. It is essential to emphasize the extent to which anxiety and depression can be deleterious for patients with SAUD. Indeed, past research showed that anxiety and depression are associated with poor treatment outcomes, as patients with SAUD presenting comorbidities double their relapse risk (Driessen et al., 2001). Preventing self-dehumanization in patients with SAUD might thus be particularly beneficial regarding symptoms of other psychopathological disorders as well as regarding their abstinence. As metadehumanization is associated with self-dehumanization, humanizing experiences might be associated with lower self-dehumanization. Humanizing patient care and providing more opportunities for patients with psychiatric disorders to have humanizing experiences outside the hospitals might be the first step to reduce self-dehumanization. However, currently, there is no validated intervention to lower self-dehumanization, and research should be conducted to this end. Developing other facets of patients’ identity that may be more humanized (e.g., being an artist or a father/mother) or reducing patients’ identification to the dehumanized group (e.g., people with SAUD) have the potential of being beneficial to their self-humanization.

The associations between metadehumanization, self-dehumanization, and psychopathological symptoms also indicate that healthcare workers and hospitals should be careful regarding how patients are being treated. Haque and Waytz (2012) argued that multiple characteristics of medicine are dehumanizing for patients. All procedures and interactions with patients before, during, and after treatment should be carefully examined to identify which parts could constitute metadehumanization sources. All of these should be optimized to reduce metadehumanization or to improve humanization, which might provide more favorable treatment conditions to patients.

### Research perspectives and limits

The model proposed in this article was developed on the most recent and relevant research in the domain. Nevertheless, the methodology used does not allow for the testing of causality, and we cannot exclude that the links between our variables might be different from those we developed (i.e., be reversed or bidirectional). Some might believe that testing the “reverse arrows” in the model would bring information in this regard, but this is not the correct way to test causality, and it does not provide meaningful information to distinguish different models (see Thoemmes, 2015). Future studies should go beyond our results, notably through longitudinal designs with repeated measures testing causal relations, in order to gain a better understanding of the dynamics of our model. Indeed, our model was built according to the current state of knowledge, but we cannot establish the causality between metadehumanization and self-dehumanization.

Research should also investigate associations between metadehumanization, self-dehumanization, and symptoms of other psychiatric disorders frequently comorbid to SAUD, such as bipolar disorders, schizophrenia, and anti-social personality disorder (American Psychiatric Association, 2013). If metadehumanization and self-dehumanization facilitate psychiatric illnesses, then improving its prevention should be a priority. Notably, developing coping strategies to reduce or prevent self-dehumanization could be beneficial for the patients. Indeed, while it is crucial to reduce the dehumanization expressed towards patients, providing them with ways to impede self-dehumanization might be a complementary strategy to protect their mental health.

## Conclusion

Experiencing dehumanization is associated with increased anxiety, depression, and drinking refusal self-efficacy in SAUD. Interestingly, self-dehumanization mediated these relations: patients reporting more metadehumanization are more likely to integrate dehumanization in their self-perception (i.e., to self-dehumanize), and this self-dehumanization mediates the links between metadehumanization and clinical outcomes. Metadehumanization and self-dehumanization are both linked to increased mood disorders’ (anxiety and depression) symptoms. Preventing metadehumanization, self-dehumanization, and promoting humanization should thus constitute a priority to improve SAUD patients’ chances of recovery.

## Data Accessibility Statements

Processed data is openly accessible on OSF (*https://osf.io/2yejv/?view_only=0f769eaf1a7343d78211017eef6e008b*).

## Additional Files

The additional files for this article can be found as follows:

10.5334/pb.1058.s1 Supplemental Material 1. Metadehumanization scale.

10.5334/pb.1058.s2 Supplemental Material 2. Self-dehumanization scale.
