# Supplementary material for: Metadehumanization and Self-dehumanization are Linked to Reduced Drinking Refusal Self-Efficacy and Increased Anxiety and Depression Symptoms in Patients with Severe Alcohol Use Disorder
Source: Psychol Belg. 2021 Jul 26;61(1):238–47. doi: 10.5334/pb.1058 (PMC8323525; doi:10.5334/pb.1058)
Supplement: Supplemental Material 1. — Metadehumanization scale. [file pb-61-1-1058-s1.pdf]

Supplementary Material 1. Metadehumanization scale.

Translated English version

Please indicate your agreement using the scale ranging from “Completely disagree” to “Completely agree”. The various levels of the scale can be used to nuance your answer.

	Completely disagree	Disagree	Slightly disagree	Neither agree or disagree	Slightly agree	Agree	Completely agree
As an alcohol-dependent person, society treats me as if I was mechanical and cold, like a robot	○	○	○	○	○	○	○
As an alcohol-dependent person, society treats me as an immature person	○	○	○	○	○	○	○
As an alcohol-dependent person, society treats me as if I was lacking empathy and sensitivity	○	○	○	○	○	○	○
As an alcohol-dependent person, society treats me as an object	○	○	○	○	○	○	○
As an alcohol-dependent person, society treats me as if I was lacking will or initiative	○	○	○	○	○	○	○
	Completely disagree	Disagree	Slightly disagree	Neither agree or disagree	Slightly agree	Agree	Completely agree

As an alcohol-dependent person, society treats me as a child	○	○	○	○	○	○	○
As an alcohol-dependent person, society treats me as if I was emotionless	○	○	○	○	○	○	○
As an alcohol-dependent person, society treats me as if I was superficial	○	○	○	○	○	○	○
As an alcohol-dependent person, society does not treat me as an individual with a personality	○	○	○	○	○	○	○
As an alcohol-dependent person, society treats me as someone lacking intelligence and competence	○	○	○	○	○	○	○
	Completely disagree	Disagree	Slightly disagree	Neither agree or disagree	Slightly agree	Agree	Completely agree
As an alcohol-dependent person, society treats me as if I was under-evolved	○	○	○	○	○	○	○
As an alcohol-dependent person, society treats me as an automaton	○	○	○	○	○	○	○
As an alcohol-dependent person, society treats me as if I was coarse	○	○	○	○	○	○	○

Original French version

Veuillez indiquer votre degré d’accord à l'aide de l'échelle allant de "Pas du tout d'accord" à "Tout à fait d'accord". Les différents niveaux de l'échelle peuvent être utilisés pour nuancer votre réponse.

	Pas du tout d'accord	Pas d'accord	Légèrement pas d'accord	Ni d'accord, ni pas d'accord	Légèrement d'accord	D'accord	Tout à fait d'accord
En tant que personne alcoolo-dépendante, la société me traite de manière mécanique et froide, comme si j’étais un robot	○	○	○	○	○	○	○
En tant que personne alcoolo-dépendante, la société me traite comme si j’étais quelqu’un d’immature	○	○	○	○	○	○	○
En tant que personne alcoolo-dépendante, la société me traite comme si j’étais une personne dénuée d’empathie et de sensibilité	○	○	○	○	○	○	○
En tant que personne alcoolo-dépendante, la société me traite comme un objet	○	○	○	○	○	○	○
En tant que personne alcoolo-dépendante, la société me traite comme si j’étais quelqu’un qui n’a pas de volonté ou d’initiative	○	○	○	○	○	○	○
	Pas du tout d'accord	Pas d'accord	Légèrement pas d'accord	Ni d'accord, ni pas d'accord	Légèrement d'accord	D'accord	Tout à fait d'accord
En tant que personne alcoolo-dépendante, la société me traite comme si j’étais un enfant	○	○	○	○	○	○	○

En tant que personne alcoolo-dépendante, la société me traite comme si j'étais un être dénué d'émotions	○	○	○	○	○	○	○
En tant que personne alcoolo-dépendante, la société me traite de manière superficielle	○	○	○	○	○	○	○
En tant que personne alcoolo-dépendante, la société ne me traite pas comme un individu doté d'une personnalité à part entière	○	○	○	○	○	○	○
En tant que personne alcoolo-dépendante, la société me traite comme si j'étais quelqu'un qui manque d'intelligence et de compétence	○	○	○	○	○	○	○
	Pas du tout d'accord	Pas d'accord	Légèrement pas d'accord	Ni d'accord, ni pas d'accord	Légèrement d'accord	D'accord	Tout à fait d'accord
En tant que personne alcoolo-dépendante, la société me traite comme si j'étais un être sous-évolué	○	○	○	○	○	○	○
En tant que personne alcoolo-dépendante, la société me traite comme si j'étais un automate	○	○	○	○	○	○	○
En tant que personne alcoolo-dépendante, la société me traite comme si j'étais quelqu'un de simple et de sommaire	○	○	○	○	○	○	○
