# Supplementary material for: Metadehumanization and Self-dehumanization are Linked to Reduced Drinking Refusal Self-Efficacy and Increased Anxiety and Depression Symptoms in Patients with Severe Alcohol Use Disorder
Source: Psychol Belg. 2021 Jul 26;61(1):238–47. doi: 10.5334/pb.1058 (PMC8323525; doi:10.5334/pb.1058)
Supplement: Supplemental Material 2. — Self-dehumanization scale. [file pb-61-1-1058-s2.pdf]

Supplementary Material 2. Self-dehumanization scale.

Translated English version

Please indicate your agreement using the scale ranging from “Completely disagree” to “Completely agree”

	Completely disagree	Disagree	Slightly disagree	Neither agree or disagree	Slightly agree	Agree	Completely agree
As an alcohol-dependent person, I consider myself to be empathic and sensitive	○	○	○	○	○	○	○
As an alcohol-dependent person, I consider myself to be a person with emotions	○	○	○	○	○	○	○
As an alcohol-dependent person, I consider myself to be a person of little intelligence and competence.	○	○	○	○	○	○	○
As an alcohol-dependent person, I consider myself to be lacking will or initiative	○	○	○	○	○	○	○
As an alcohol-dependent person, I sometimes consider myself as an automaton	○	○	○	○	○	○	○
As an alcohol-dependent person, I consider myself to be coarse	○	○	○	○	○	○	○

	Completely disagree	Disagree	Slightly disagree	Neither agree or disagree	Slightly agree	Agree	Completely agree
As an alcohol-dependent person, I consider myself to be an individual with a personality	○	○	○	○	○	○	○
As an alcohol-dependent person, I sometimes act like a child	○	○	○	○	○	○	○
As an alcohol-dependent person, I sometimes feel mechanical and cold, like a robot	○	○	○	○	○	○	○
As an alcohol-dependent person, I sometimes consider myself to be an inanimate object	○	○	○	○	○	○	○
	Completely disagree	Disagree	Slightly disagree	Neither agree or disagree	Slightly agree	Agree	Completely agree
As an alcohol-dependent person, I consider myself to be superficial	○	○	○	○	○	○	○
As an alcohol-dependent person, I sometimes feel like I am immature	○	○	○	○	○	○	○
As an alcohol-dependent person, I sometimes consider myself as an under-evolved	○	○	○	○	○	○	○

being							

Original French version

Veuillez indiquer votre degré d’accord à l'aide de l'échelle allant de "Pas du tout d'accord" à "Tout à fait d'accord"

	Pas du tout d'accord	Pas d'accord	Légèrement pas d'accord	Ni d'accord, ni pas d'accord	Légèrement d'accord	D'accord	Tout à fait d'accord
En tant que personne alcoolo-dépendante, j'estime être une personne dotée d'empathie et de sensibilité	○	○	○	○	○	○	○
En tant que personne alcoolo-dépendante, j'estime être une personne dotée d'émotions	○	○	○	○	○	○	○
En tant que personne alcoolo-dépendante, je me considère comme quelqu'un de peu intelligent et peu compétent	○	○	○	○	○	○	○
En tant que personne alcoolo-dépendante, je considère faire preuve de peu de volonté ou d'initiative	○	○	○	○	○	○	○
En tant que personne alcoolo-dépendante, j'ai parfois l'impression d'agir comme un	○	○	○	○	○	○	○

automate							
En tant que personne alcoolo-dépendante, je me vois comme quelqu'un de simple et de sommaire	○	○	○	○	○	○	○
	Pas du tout d'accord	Pas d'accord	Légèrement pas d'accord	Ni d'accord, ni pas d'accord	Légèrement d'accord	D'accord	Tout à fait d'accord
En tant que personne alcoolo-dépendante, j'estime être un individu doté d'une personnalité à part entière	○	○	○	○	○	○	○
En tant que personne alcoolo-dépendante, j'ai parfois l'impression de me comporter comme un enfant	○	○	○	○	○	○	○
En tant que personne alcoolo-dépendante, j'ai parfois l'impression de me comporter de manière mécanique et froide, comme si j'étais un robot	○	○	○	○	○	○	○
En tant que personne alcoolo-dépendante, je me perçois parfois comme un objet inanimé	○	○	○	○	○	○	○

	Pas du tout d'accord	Pas d'accord	Légèrement pas d'accord	Ni d'accord, ni pas d'accord	Légèrement d'accord	D'accord	Tout à fait d'accord
En tant que personne alcoolo-dépendante, j'ai le sentiment d'être quelqu'un de superficiel	○	○	○	○	○	○	○
En tant que personne alcoolo-dépendante, j'ai le sentiment d'être quelqu'un d'immature	○	○	○	○	○	○	○
En tant que personne alcoolo-dépendante, je me considère parfois comme un être sous-évolué	○	○	○	○	○	○	○
